# Spontaneous rupture of the eyeball due to choroidal metastasis of gastric carcinoma: A case report

**DOI:** 10.1097/MD.0000000000017441

**Published:** 2019-10-04

**Authors:** Shuang-Qing Wu, Qiu-Shi Li, Yu Zhang, Li-Wei Zhu

**Affiliations:** Department of Ophthalmology, Zhejiang Medicine and Western Medicine Integrated Hospital (Hangzhou Red-Cross Hospital), Hangzhou, Zhejiang, P.R. China.

**Keywords:** choroid metastasis, enucleation, gastric carcinoma, spontaneous rupture

## Abstract

**Rationale:**

Although metastatic tumor is the most common intraocular malignancy, choroidal metastasis from gastric cancer is relatively rare. We present the exact course of a spontaneous rupture of the eyeball with choroidal metastasis from gastric carcinoma (GC) and the applied surgical interventions.

**Patient concerns:**

A 59-year-old male presented with pain and vision loss on his left eye for 6 months. He was diagnosed with GC, for which he received systemic chemotherapy for a year.

**Diagnoses:**

Preoperative B-scan, color fundus photography, computed tomography, and magnetic resonance imaging showed a choroidal tumor in his left eye. The spontaneous rupture of the corneoscleral limbus from 2 to 5 o’clock, combined secondary glaucoma, exudative retinal detachment and choroidal detachment were found.

**Interventions:**

The ruptured corneoscleral limbus was sutured and the orbit was lavaged with 0.4% cisplatin during the enucleation.

**Outcomes:**

Histopathology confirmed high homology of the choroidal metastasis and GC. He survived for 2 months after surgery, without pain or orbital neoplasms.

**Lessons:**

Choroidal metastasis from GC rapidly progressed to spontaneous rupture of the eyeball. Careful eyeball enucleation followed by orbital lavage with chemotherapeutics may reduce metastasis risk beyond the eyeball. Additional therapeutic interventions should be considered in patients resistant to single systemic chemotherapy.

## 1 Introduction

Metastatic tumor is the most common type of intraocular malignancy. The uveal tissue, particularly of the choroid, on account of its high blood flow, is the most favored site of metastasis.^[1]^ Not surprisingly, the choroid accounts for 62% to 88% of all ocular metastasis, followed by the iris and ciliary body.^[2–4]^ The most common primary site of malignancy is the breast (16%–68%) and lungs (21%–50%).^[1,3,5]^ Choroidal metastases originating from the gastrointestinal tract have been less frequently reported (4%, 18/520 eyes).^[3,6]^ Gastric carcinoma (GC) develops slowly with the possibility of the development of multiple metastases in the liver and peritoneum,^[7,8]^ whereas ocular metastasis is rare.^[1,6,9]^ Thus, there is a lack of systematic analyzes limited to only GC related choroidal metastases. Here, we have presented the case of a patient with choroidal metastasis of GC, in whom the corneoscleral limbus spontaneously ruptured, despite systemic chemotherapy, due to uncontrolled disease course.

## 2 Case presentation

**Figure 1:**
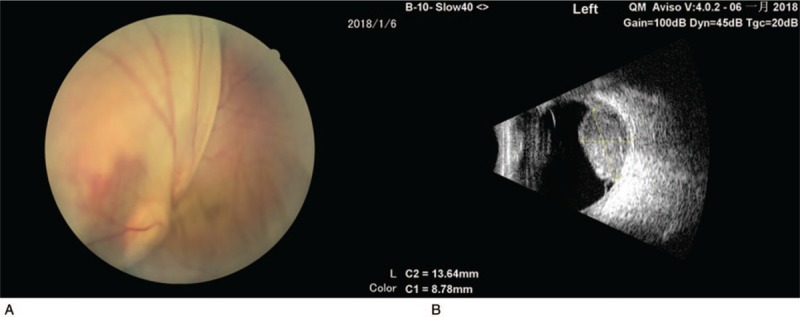
(A) Color fundus photograph of the left eye reveal a nasal choroidal mass with associated serous retinal detachment; (B) B-scan ultrasonography of the left eye demonstrates a choroidal lesion measuring 8.78 × 13.64 mm with high reflectivity.

A retrospective chart review of the patient was conducted at the Hangzhou Red-Cross Hospital, after obtaining approval by the Institutional Review Board. A 59-year-old male with GC was referred to our institution with pain and decreased vision in his left eye, over a 6-month period after being diagnosed in another hospital with an intraocular tumor and secondary retinal detachment according to B-scan and color fundus photographs (Fig. 1). A year prior, he was diagnosed as GC and femoral metastasis, staged as T3N3M1. Instead of surgery, he underwent systemic chemotherapy, including intravenous oxaliplatin 85 mg/m^2^ once and oral tegafur 100 mg/d for 14 consecutive days per 3 weeks and erbitux 250 mg/m^2^ intravenously per 3 weeks for targeted therapy. Oxycontin was used to alleviate pain. He denied any history of either ocular disease or intraocular surgery.

**Figure 2:**
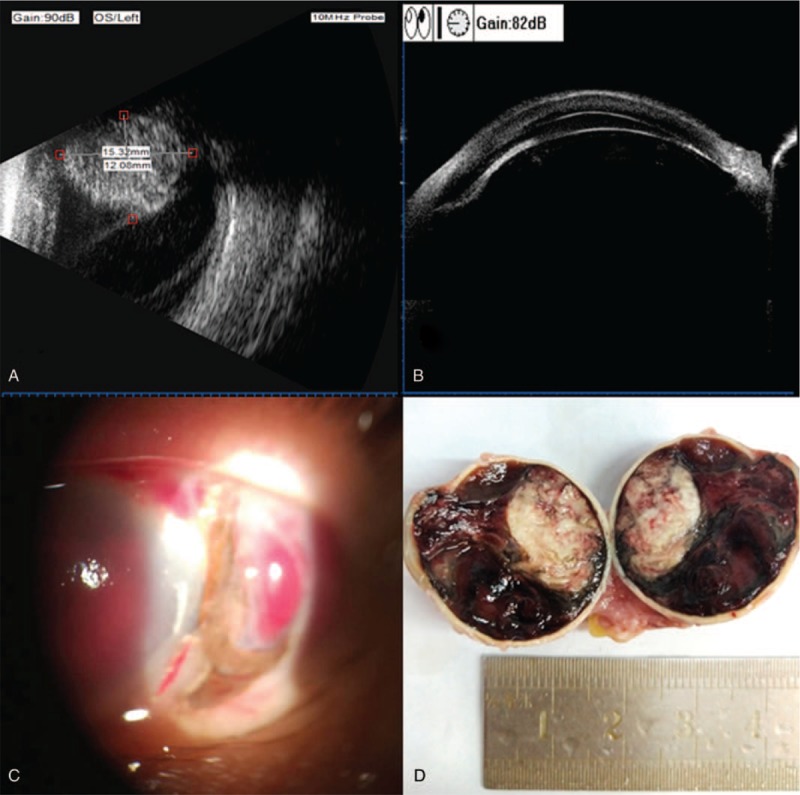
(A) B-scan ultrasonography of the left eye demonstrated a choroidal lesion measuring 12.08 × 15.32 mm with high reflectivity and extensive exudative retinal detachment; (B) Ultrasound biomicroscopy showed the squeezed anterior chamber with hyphemia; (C) Spontaneous rupture of the corneoscleral limbus from 2 to 5 o’clock, edema of cornea, and very shallow anterior chamber with hyphemia; (D) Nasal-posterior solid neoplasm was found in the enucleated eyeball, with massive hematocele.

The patient presented with total loss of vision in his left eye. The anterior segment revealed mixed-congestion and edema of conjunctiva, rupture of corneoscleral limbus from 2 to 5 o’clock, edema of cornea, very shallow and squeezed central anterior chamber with complete loss of peripheral anterior chamber detected by ultrasound biomicroscopy, and hyphemia (Fig. 2). The posterior segment was indistinguishable. A nasal-posteriorly located choroidal tumor measuring 12.08 × 15.32 mm was detected by B-scan ultrasonography, combined with exudative retinal and choroidal detachment (Fig. 2).

**Figure 3:**
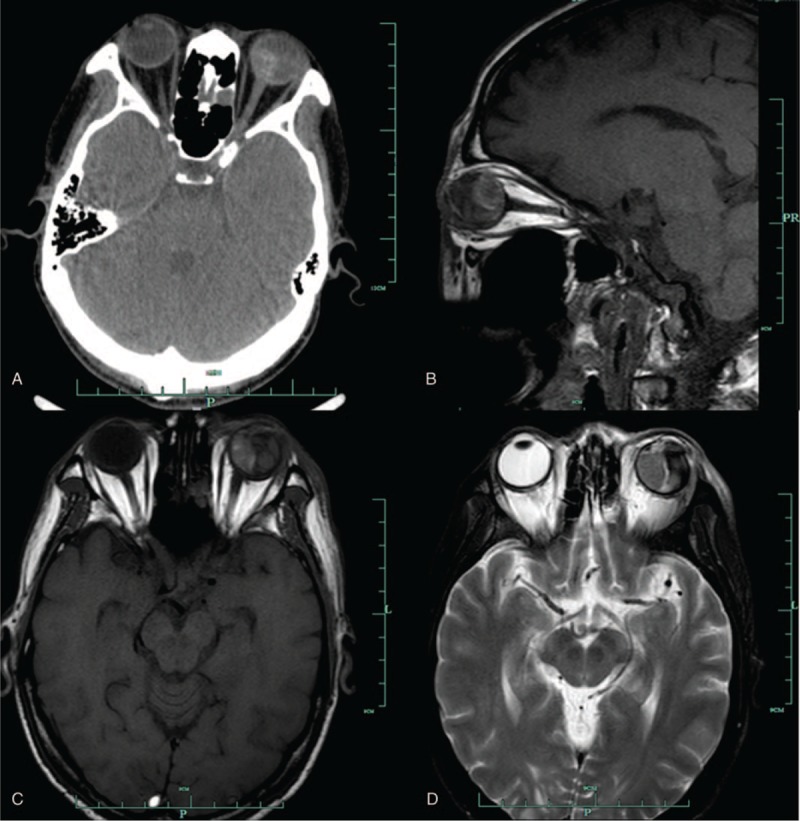
(A) Computed tomography scan showing an enhanced mass at the nasal and posterior side of the left eye; (B) MRI scan showing the same lesion on the left eye; (C) The T1-weighted image shows isointensity with enhancement; (D) The T2-weighted image shows hypointensity. MRI = magnetic resonance imaging.

A computed tomography (CT) scan of the orbits showed an enhanced mass at the nasal and posterior side of the choroid of the left eyeball (Fig. 3). Magnetic resonance imaging (MRI) depicted the same lesion as an isointense signal on the T1-weighted image and as a hypointense signal on the T2-weighted image (Fig. 3). Based on the patient's history and our findings, left choroidal metastasis from GC was impressed.

**Figure 4:**
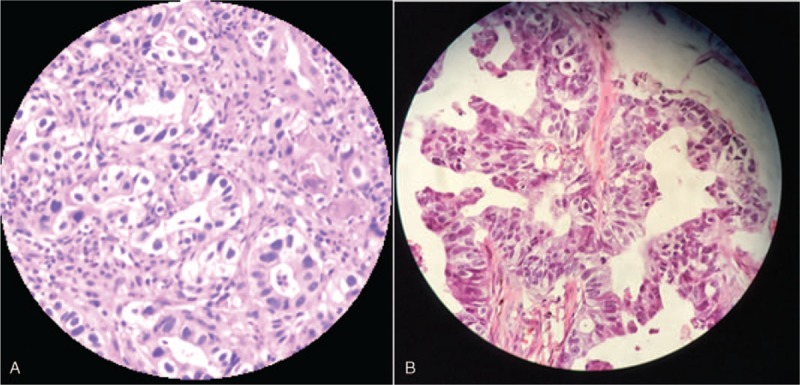
(A) Endoscopic biopsy specimen from the stomach showing well-differentiated tubular adenocarcinoma (H&E staining, original magnification ×100); (B) Enucleated specimen of the left eye showing a moderately differentiated tubular adenocarcinoma (H&E staining, original magnification ×100). H&E = hematoxylin-eosin.

Subsequently, the patient underwent enucleation. To reduce the risk of orbital metastasis, we sutured the ruptured corneoscleral limbus in advance and used 0.4% cisplatin solution to lavage the orbit after enucleation during surgery. Nasal-posterior solid neoplasm was found in the enucleated eyeball, accompanied by massive hematocele (Fig. 3). The choroidal metastasis and GC showed significant correlation by histopathology (Fig. 4). The patient was relieved of pain and recovered well from surgery. He did not complain of neoplasm or eye pain at follow-up visits at 1 and 4 weeks, but died 2 months after surgery.

## 3 Discussion

Gastric cancer is one of the most common causes of cancer-related mortality in the world. The ocular metastasis of gastric cancer is most often located on the choroid,^[3]^ followed by the iris,^[10,11]^ eyelid and conjunctiva,^[12]^ optic nerve,^[13]^ and skin,^[7]^ as shown in a few case studies.

Choroid metastasis manifests as blurred vision (70%–81%), flashes and floaters (5%–12%), eye pain (5%–14%), and 9% to 11% of patients do not exhibit any symptoms.^[1,3,9]^ Patients with choroid metastases from GC most often complain of visual disorder, due to early onset of macular edema, which was associated with accumulation of subretinal fluid and involvement of the macular region.^[7,14–17]^ At the initial visit, the main complaint of our case was eye pain, which was probably related with secondary glaucoma, iridocyclitis, and scleral invasion of the tumor. Gradual decrease in visual acuity occurred earlier than eye pain in our patient, and his negative attitude to treatment delayed his first visit to the ophthalmology clinic. Eventually, massive hemorrhage and increased intraocular pressure caused spontaneous rupture of the corneoscleral limbus in the eyeball.

Choroid metastasis usually manifests as a yellow subretinal mass (94%) accompanied with subretinal fluid (73%)^[3]^ and choroid metastasis of GC is similar to that of the lung and breast,^[1,9]^ as presented by our patient at his first ophthalmologic visit. Choroidal metastasis from GC has been reported previously, with macular edema being a common feature, besides exudative retinal detachment and subretinal hemorrhage.^[7,14–17]^ In fact, choroid metastasis usually exhibits a higher incidence of exudative retinal detachment compared to uveal melanomas.^[18]^ Ultrasonography revealed a choroidal mass with moderate-to-high internal reflectivity. A well-circumscribed subretinal metastatic carcinoma, appearing as an isointense signal on the T1-weighted image and as a hypointense signal on the T2-weighted image^[19]^ was revealed by MRI. In addition, optical coherence tomography examination shows irregular elevation of choroidal tissue and aids in early diagnosis.^[20]^ Based on the history of GC, manifestations of fundoscopy, and the results of ultrasonography, CT, and MRI, our patient was diagnosed with choroidal metastasis from GC, which was confirmed by histopathology after surgery.

Possible treatment options for choroidal metastasis include systemic chemotherapy, intravitreal injection of anti-vascular endothelial growth factor injections, localized radiation, transpupillary thermotherapy, photodynamic therapy, and surgical resection.^[21–24]^ Radiotherapy and photocoagulation were the most common treatments due to photosensitivity of the choroid.^[1,9]^ However, systemic chemotherapy is commonly used for choroidal metastasis from GC as it is insensitive to radiotherapy.^[3]^ A combination of systemic paclitaxel and local intensity-modulated radiotherapy provided remission in a case of choroidal metastasis from GC,^[15]^ while chemotherapy with oral S-1 and intravenous cisplatin reduced choroidal lesions in another case.^[7]^ However, choroidal metastasis from GC was found to be resistant to systemic imatinib.^[16]^

Choroidal metastasis of our patient occurred 6 months after GC was diagnosed. Although the patient received systemic chemotherapy with oxaliplatin, tegafur, and erbitux, choroidal metastasis in the patient progressed, inducing spontaneous rupture of the eyeball. While our enucleation procedure reduced the risk of orbital recurrence of the tumor, Melo et al^[25]^ enucleated the affected eye when choroidal tumor from GC infiltrated the macular area and extended around the optic nerve head, orbital recurrence still occurred. Indeed, no orbital metastasis-related symptoms were reported by our patient during the 2-month postoperative follow-up. The patients with choroidal metastasis have a poor prognosis.^[26,27]^ The reported cases with choroidal metastasis from GC had survival durations of 9 months^[16]^ and 3 years,^[15]^ and our patient died 8 months after the first diagnosis of choroidal metastasis. Poor response to chemotherapy and long duration of choroidal metastasis from GC may have led to spontaneous rupture of the eyeball.

The case of a patient with choroidal metastasis from GC was presented. The rapid progression led to spontaneous rupture of the eyeball. Careful enucleation of the eyeball followed by lavage of the orbit with chemotherapy drugs may reduce the risk of metastasis beyond the eyeball structure.

## Author contributions

**Conceptualization:** Qiu-Shi Li.

**Data curation:** Shuang-Qing Wu, Qiu-Shi Li, Yu Zhang.

**Funding acquisition:** Shuang-Qing Wu.

**Investigation:** Qiu-Shi Li, Liwei Zhu.

**Methodology:** Liwei Zhu.

**Resources:** Shuang-Qing Wu, Yu Zhang, Liwei Zhu.

**Supervision:** Liwei Zhu.

**Writing – original draft:** Shuang-Qing Wu, Qiu-Shi Li.

**Writing – review & editing:** Shuang-Qing Wu, Liwei Zhu.
